# Preference of specimen collection methods for human papillomavirus detection for cervical cancer screening: a cross-sectional study of high-risk women in Mombasa, Kenya

**DOI:** 10.1186/s12978-018-0651-z

**Published:** 2018-12-12

**Authors:** Griffins O. Manguro, Linnet N. Masese, Kishor Mandaliya, Susan M. Graham, R. Scott McClelland, Jennifer S. Smith

**Affiliations:** 1 Kenyatta National Hospital, P.O. Box 91109, Mombasa, 80103 Kenya; 2 Department of Epidemiology, University of Washington, P.O Box 357236, Seattle, WA, 98195 USA; 3 Pathcare Laboratories, Mombasa, Kenya; 4 Departments of Epidemiology, Global Health, and Medicine, University of Washington, P.O Box 359909, Seattle, WA USA; 5 Institute of Tropical and Infectious Diseases, University of Nairobi, Nairobi, Kenya; 6 University of North Carolina at Chapel Hill, Chapel Hill, NC USA; 7 Lineberger Cancer Center, Chapel Hill, NC USA

**Keywords:** HrHPV, Self-collection, Clinician-collection, Cervical cancer, Screening, Kenya

## Abstract

**Objectives:**

Self-collection of genital specimens for high-risk types of human papillomavirus (hrHPV) detection may increase cervical cancer screening uptake. We hypothesized that women would prefer self-collection to clinician-collection of genital specimens. To test this hypothesis, and women’s preference between two different self-collection approaches, a total of 199 women were enrolled in a cross-sectional study in Mombasa, Kenya.

**Materials and methods:**

Participants provided self-collected specimens using the Evalyn cytobrush (Rovers) stored in a dry tube and the Viba cytobrush (Rovers) stored in wet Aptima media (Hologic). A clinician also collected cervical specimens for hrHPV testing and for cytology, and performed visual inspection using acetic acid. A post-examination questionnaire assessed preferences for the different methods of specimen collection. To test the difference in proportions for each collection method, we performed an exact binomial probability test, under the null hypothesis that women would prefer each specimen-collection method equally.

**Results:**

Most women preferred clinician-collection over self-collection (68% versus 32%, *p* < 0.01). For self-collection, dry-self collection with the Evalyn brush was preferred over the wet-selection with the Viba brush (53% versus 27%, p < 0.01). There was no association between preference for self-collection and preference for a particular self-collection cytobrush.

**Conclusion:**

Further research to understand and address obstacles to self-collection of genital specimens may be needed to improve the uptake of self-collection for cervical cancer screening, especially in settings with poor access to trained healthcare providers.

## Plain English summary

Testing for high-risk sub-types of human papillomavirus (hrHPV) is an effective way of screening for cervical cancer. Unlike Pap smears which have to be performed by a clinician, women can self-collect genital samples for HPV testing. Such samples often produce results that are as accurate as those obtained from clinician-collected samples.

Between August 2013 and April 2015, we conducted a study among 199 women at high risk of sexually transmitted infections (STIs) and HIV in Mombasa, Kenya. Our aim was to find out if the women preferred to collect their own genital samples or have samples collected by a clinician, and between two self-collection brushes, which one they would like most. Study participants performed self-collection in a private room, followed by clinician-collection and a pap smear.

More than two thirds (68%) of the women preferred to have a clinician collect the genital samples. For self-collection, majority of women (53%) preferred the Evalyn brush, which was stored and transported without any preservative (dry) compared to the Viba brush, which was stored and transported in a liquid preservative.

Further research is needed to find out reasons why women in this setting prefer to have genital samples collected by a clinician rather than to collect samples themselves, considering that self-collected vaginal samples is likely to be easier, more comfortable, less embarrassing and just as accurate as those collected by a clinician. Understanding such factors may help to scale-up self-testing, which may increase the number of women who screen for cervical cancer.

## Background

Detection of high-risk human papillomavirus (hrHPV) in genital specimens is current being used in conjunction with (co-testing) or as an alternative to Papanicolau (Pap) smears for primary cervical cancer screening [1–6]. Testing for hrHPV in genital specimens has demonstrated significantly greater sensitivity as compared to conventional Pap smear for detecting high-grade cervical intra-epithelial neoplasia (CIN 2+) [2, 6, 7]. Additionally, women can self-collect cervico-vaginal specimens for hrHPV testing. These self-collected specimens demonstrate similar sensitivity and specificity to clinician-collected specimens for detecting high-grade cervical lesions (CIN 2+), and may improve access to screening [8]. Studies conducted in Europe, North and South America looking at the acceptability of self-collection of genital specimens for hrHPV testing indicate that self-collected HPV genital specimens were preferred compared to the conventional Pap smears for HPV screening. In the latter one study from South America, age and level of education were strongly correlated with increased preference for self-collection [4, 9]. Few studies [10, 11] have explored the acceptability of self-collection of genital specimens in Africa, where cultural and religious beliefs concerning both clinician and self-collection of genital specimens may be an important consideration. Understanding women’s preference for self-collection versus clinician-collection of specimens will help to guide future interventions for cervical cancer screening.

Our study, conducted in a cohort of high-risk women in Mombasa, Kenya, assessed women’s preference for self-collection versus clinician-collection of genital specimens for detection of hrHPV for primary cervical cancer screening. We also explored women’s preference between two different self-collection devices, the Viba cytobrush (Rovers®, Netherlands), which required a liquid transport media, and the Evalyn cytobrush (Rovers®, Netherlands), which did not. Based on existing data, we hypothesized that women would prefer self-collection over clinician collection [9]. Due to the presumed ease of collection, we also hypothesized that Evalyn cytobrush (dry) would be preferred over the Viba cytobrush (in liquid media) for self-collection of specimens.

## Materials and methods

### Study design

We conducted a clinic-based, cross-sectional study from August 2013 to April 2015. A total of 200 women participating in a cohort study of women at high risk of acquiring sexually transmitted infections (STIs) and HIV in Mombasa, Kenya were enrolled. Clinical procedures, including self-collection of hrHPV specimens, were performed at the Ganjoni Health Centre in Mombasa. Study procedures were integrated into the ongoing follow-up procedures for the Mombasa Cohort [12, 13]. Briefly, the Mombasa Cohort is an open cohort study of female sex workers (FSWs) established in 1993 as a HIV incidence cohort. FSW are recruited from major sex work venues in Mombasa. Those interested are referred to the research clinic, located in the Ganjoni Municipal Clinic. Women are invited into this parent cohort if they are 18–45 years old, reside in Mombasa area, are self-identifying as exchanging sex for payment in cash or in kind and are able to provide informed consent. Routine cohort visits are scheduled every month. Every 3 months, the scheduled visit procedures include collection of genital specimens for *Chlamydia trachomatis* and *Neisseria gonorrhea* nucleic acid amplification testing (NAAT). For this ancillary study, we invited women whose study visits coincided with the NAAT specimen collection visit. We used convenience sampling to enroll those who agreed to participate from among those who were eligible.

### Study visit procedures

A study nurse briefly introduced the study to eligible women and invited them to participate. Women who agreed to participate were referred to the study counselor, who provided further information and responded to questions. Following this discussion with the counselor, interested participants were required to provide written informed consent.

**Fig. 1:**
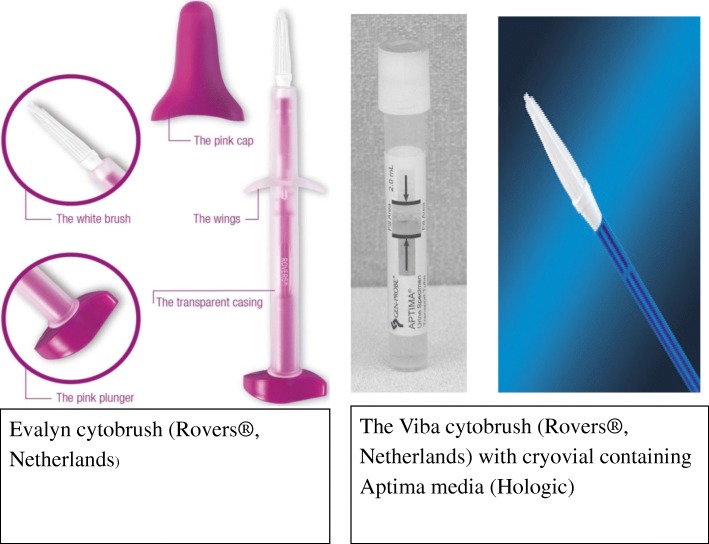
The Evalyn cytobrush (left side) and the Viba cytobrush with cryovial containing Aptima media (right side)

Self-collection of genital specimens was conducted in a private room at the health facility. A study nurse provided verbal instructions on self-collection procedures, after which participants performed the procedure on their own. Pictorial diagrams with detailed instructions on self-collection were also available in the room. Each woman performed self-collection using the two different specimen brushes, the Evalyn cytobrush (Rovers®, Netherlands), and the Viba cytobrush (Rovers®, Netherlands). To minimize potential bias from the order of specimen collection, women had study numbers assigned to them based on their enrollment into the study, and those with odd study numbers self-collected using the Evalyn brush first, while those with even study numbers self-collected using the Viba cytobrush first. The physical characteristics and specimen handling slightly differ between these two devices (Fig. 1). For both cytobrushes, self-collected specimens were obtained by inserting the collection brush and following the manufacturer’s instructions to sample cells from the cervico-vaginal wall.

The Evalyn cytobrush is pink in color, and consists of a plunger containing white bristles made of polyethylene at the tip, a transparent casing, and a transparent cap. For self-collection, women were instructed to squat, pull the plunger down into the clear casing to protect the bristles, and gently insert the brush into the vagina. Next, they pushed the plunger back up to expose the bristles, and rotated the brush through five complete turns in the same direction for specimen collection. Each complete rotation was accompanied by an audible click on the device. Self-collection using the Evalyn brush was “dry,” in that there was no need to place the specimens in a preservative for transportation to the laboratory.

The Viba brush consists of a blue handle and an easily removable white tip. The tip contains bristles made of similar material as the Evalyn brush. The Viba brush was provided with a cryovial containing 1 ml of Aptima media (Hologic®, USA). After self-collection, participants broke off the brush head at the tip of the cytobrush and deposited it into the cryovial containing Aptima media.

After self-collection, a study clinician performed a speculum-assisted pelvic examination, which included collection of cervical specimen for hrHPV testing. Clinician-collection of specimens was performed using a cervical specimen collection brush (Hologic®, USA). Unlike self-collected specimens, clinician specimens were collected from the endocervix. Similar to the Viba brush, clinician-collected specimens were stored in Aptima media (Hologic®, USA). After hrHPV specimen collection, the clinician performed visual inspection under acetic acid and a conventional Pap smear. All clinicians in this study had extensive training and experience in genital examination and specimen collection as part of the procedures in the Mombasa Cohort.

### Post-examination questionnaire to assess women’s preference

Following the clinical examinations, women participated in a structured interview using a standardized questionnaire to assess their preferences for different methods of specimen collection. For the question on self-collection versus clinician-collection, women were asked, “If you were to be tested for hrHPV infection again, how would you like the specimen to be collected?” They gave their answers as either self-collection or clinician-collection.

To assess women’s preferences between the Evalyn and Viba cytobrushes, they were asked, “Which of the two brushes you collected yourself did you like best?” They were then asked to select one of three possible responses; “I liked the blue brush (Viba brush),” “I liked the pink brush (Evalyn brush),” and “I liked them both equally”. To explore women’s perceptions of specific characteristics of the two brushes, they were asked four questions. Their responses to these questions were graded on a 5-point Likert scale, with a range of one for “strongly agree”, two for “agree somewhat”; three for “neutral”; four for “disagrees somewhat”; and five for “strongly disagree.” The four questions were: “Was the brush comfortable to insert?” “Were you concerned about hurting yourself with the brush?”, “Were you concerned about using the brush properly?”, and, “Were the instructions for self-collection using the brush easy to understand?”

### Statistical analysis

The primary aim of the study was to test the hypothesis that women would prefer self-collection to clinician-collection of genital specimens. To test the difference in proportions for each collection method, we performed an exact binomial probability test, under the null hypothesis that women would prefer each specimen-collection method equally (i.e., *p* = 0.50 for selecting self-collection or clinician-collection). To determine which self-collection cytobrush was most preferred, we compared the proportion of women who preferred the Evalyn brush to those who preferred the Viba brush and to those who preferred them both equally. To determine the statistical significance of this difference, we performed a one-sample t-test, with responses distributed between 0 for the Viba brush, 1 for no preference, and 2 for the Evalyn brush, testing the null hypothesis of no difference in preference (i.e., mean preference = 1.0).

Log-binomial regression analyses were performed to explore the predictors of preference for clinician collection versus self-collection. The variables tested were identified a priori through a review of the literature. We included factors associated with self-testing for other conditions (HIV) and those associated with screening for cervical cancer. These included women’s age at first sexual intercourse [14], current age [15], level of education [16, 17], marital status [16], parity [15], and religion [18].

To explore predictors of preference for the dry self-collection brush, wet self-collection brush, and preference for both equally, we performed a multinomial logistic regression analysis, with no preference as the referent category. The predictors included in the models were similar to those included in our analysis of predictors of preference for self-collection versus clinician-collection. Relative risk ratios greater than 1 in this analysis indicated a higher probability of preferring the brush specified, relative to having no preference. Conversely, relative risk ratios less than 1 indicated a lower probability of preferring the brush specified, relative to having no preference. We performed chi-squared tests to explore whether there was an association between preference for self-collection and preference for a particular collection device. Finally, to better understand women’s preferences for specific collection devices, we explored their perceptions about characteristics of the two brushes. Median and interquartile ranges were used to summarize the responses to each question that was graded on a Likert scale. Wilcoxon signed-ranks tests were performed to analyze differences in paired responses to each of these questions for the Evalyn brush compared to the Viba brush. All analyses were performed using STATA version 13.

## Results

Between August 2013 and April 2014, a total of 200 women were enrolled into the cervical cancer screening study. Of these, 199 (99%) participated in the post-examination interview and were included in analyses. Mean age of study participants was 37.6 years, range (30–44) and approximately half were HIV-positive (Table 1).

**Table 1:** Baseline characteristics of 199 female sex workers from Mombasa, Kenya

Characteristic	Mean (SD) or Number (percent)
Age (years)	37.6 (9.5)
Age at first sex (years)	16.9 (2.3)
> 8 years of education (At least some high-school)	91 (46%)
Ever pregnant	183 (92%)
Ever married	120 (60%)
Religion	
Christian	176 (89%)
Muslim	18 (9%)
Other^a^	5 (3%)
Using modern contraception other than condoms alone	
No contraceptive method	63 (32%)
Hormonal contraceptive use	53 (27%)
Non-hormonal contraceptive use	83 (41%)
HIVseropositive	101 (51%)

^a^Includes traditional African religions and no religious affiliation

Of 199 women, 63 (32%) reported preference for self-collection compared to 136 (68%) who reported preference for clinician-collection (exact binomial probability test, *p* < 0.001). Baseline demographic and clinical characteristics were broadly similar between women who preferred clinician-collection and those who preferred self-collection. Univariate log-binomial regression analyses of predictors of preference for self-collection found that older age at sexual debut was significantly associated higher with preference for clinician-collection versus self-collection (odds ratio [OR] 1.10, 95% confidence interval [CI] 1.02–1.10, *p* = 0.02) (Table 2).

**Table 2:** Univariate log-binomial regression analyses exploring predictors of women’s preference for clinician-collection versus self-collection of genital specimens

Variable	Overall Number (Percent) of women who prefer self-collection *N* = 199	Women who prefer clinician-collection *n* = 136	Women who prefer self-collection *n* = 63	Relative Risk (95% CI) for preference of clinician versus self-collection	*p*-value
		Mean (SD) or Number (Percent)	Median (IQR) or Number (Percent)		
Age (years)		38.2 (9.7)	36.4 (9.1)	0.99 (0.97–1.0)	0.25
Age at first sex (years)		16.6 (2.2)	17.4 (2.4)	1.10 (1.02–1.19)	0.02
Parity					
Nulliparous	44%	9 (7%)	7 (11%)	Reference	
Ever pregnant	31%	127 (93%)	56 (89%)	0.71 (0.39–1.28)	0.26
Education					
8 years and less	27%	79 (58%)	29 (46%)	Reference	
v > 8 years	37%	57 (42%)	34 (54%)	1.39 (0.92–2.10)	0.11
Marital status					
Never married	30%	55 (40%)	24 (38%)	Reference	
Ever married	33%	81 (60%)	39 (62%)	1.07 (0.70–1.63)	0.75
HIV status					
HIV-negative	35%	64 (47%)	35 (56%)	Reference	
HIV-positive	28%	72 (53%)	28 (44%)	0.79 (0.52–1.20)	0.27
Religion					
Christian		122 (90%)	54 (86%)	Reference	
Muslim		18 (9)	8 (13%)	1.45 (0.83–2.54)	0.20
Other^a^		5 (3)	1 (2%)	0.65 (0.11–3.82)	0.64

^a^Traditional African religions and no religious affiliation

For self-collection, the Evalyn brush was generally preferred (*n* = 105; 53%), compared to the Viba brush (*n* = 50; 25%), and equal preference for both brushes (*n* = 44; 22%) (One-sample t test *p* < 0.001). We found no association between preference for specimen collection method self-versus clinician) and preference for self-collection cytobrush (Chi-square test, *p* = 0.69). In univariate multinomial logistic regression analyses, age, parity, comfort when using the brush and concerns of getting hurt while using the brush were significantly associated with preference for a particular self-collection method (Table 3). In multivariable analyses, increased comfort with the Evalyn brush was associated with lower probability of selecting the Viba brush (OR 0.48, 95% CI 0.32–0.71), and increased concerns about hurting themselves with the Viba brush were associated with an increased probability of selecting the Evalyn brush (OR 1.33, 95% CI 1.05–1.71).

**Table 3:** Multinomial regression analysis exploring predictors of women’s preference for the Viba or the Evalyn cytobrush

Variable	Prefers Viba cytobrush		Prefers Evalyn cytobrush	
	Relative risk ratio (95% CI)	*p*-value	Relative risk ratio (95% CI)	*p*-value
Age (years)	0.92 (0.88–0.97)	0.001	0.94 (0.91–0.98)	0.004
Age at first sex (years)	1.07 (0.89–1.28)	0.47	1.05 (0.90–1.23)	0.55
Parity				
Nulliparous	Reference		Reference	
Ever pregnant	0.68 (0.52–0.90)	0.008	0.82 (0.65–1.02)	0.07
Education				
No high school education	Reference		Reference	
At least high school-level	1.22 (0.54–2.78)	0.63	1.42 (0.70–2.88)	0.34
Marital status				
Never married	Reference		Reference	
Ever married	0.81 (0.35–1.85)	0.62	0.95 (0.46–1.95)	0.88
HIV status				
HIV-negative	Reference		Reference	
HIV-positive	1.16 (0.51–2.63)	0.72	0.62 (0.31–1.26)	0.19
Religion				
Christian	Reference		Reference	
Muslim	0.35 (0.07–1.93)	0.23	0.92 (0.30–2.83)	0.89
Other^a^	2.66 (0.27–26.62)	0.41	0.42 (0.03–6.87)	0.54
Evalyn brush more comfortable to insert^b^	0.64 (0.46–0.89)	0.009	1.38 (0.92–2.06)	0.12
Viba brush more comfortable to insert ^b^	1.48 (1.01–2.16)	0.04	0.69 (0.55–0.87)	0.002
Concerned about hurting themselves with Evalyn brush ^b^	1.25 (0.98–1.58)	0.07	0.89 (0.72–1.09)	0.25
Concerned about hurting themselves with Viba brush ^b^	0.93 (0.74–1.18)	0.56	1.38 (1.12–1.69)	0.002

^a^Traditional African religions and no religious affiliation

^b^For this analysis, the rating scale for these questions was reverse coded (5 strongly agree, 4 agree somewhat, 3 neutral, 2 disagree somewhat, 1 strongly disagree) so that increasing ratings indicated agreement with each item and higher relative risk ratios indicated a higher probability of preferring the brush specified

Table 4 presents responses to questions comparing women’s experience with the Evalyn and Viba cytobrushes, together with *p*-values from the test for differences in paired responses to each of these questions. Participants strongly agreed that instructions for both self-collection brushes were easy to understand. In terms of analyses comparing women’s experience with the Evalyn and the Viba cytobrushes, for the questions, “Were the instructions for self-collection using the brush easy to understand?” and, “Were you concerned about using the cytobrush properly?” women rated both self-collection cytobrushes equally and favorably. For the question “Were you concerned about hurting yourself with the brush?” responses were more favorable for the Evalyn brush as compared to the Viba brush. For the question “Was the brush comfortable to insert?” a significantly greater proportion of women reported comfort with the Evalyn brush compared to the Viba brush.

**Table 4:** Median scores and interquartile range for responses to questions on experience when using the cytobrushes and *p*-values from the Wilcoxon signed ranks test for differences in each paired response

Question	Evalyn	Viba	*p* value
	Median (IQR)	Median (IQR)	
Were the instructions on using brushes easy to understand?	1 (1–1)	1 (1–1)	0.08
Were you concerned about using the brush properly?	2 (1–4)	2 (1–4)	0.15
Were you concerned about hurting yourself?	4 (1–5)	2 (1–5)	0.0004
Was the brush comfortable to insert?	1 (1–1)	1 (1–4)	< 0.001

(1 strongly agree, 2 agree somewhat, 3 neutral, 4 disagree somewhat, 5 strongly disagree)

## Discussion

In this study of 199 high-risk Kenyan women participating in an HIV incidence cohort, significantly more participants preferred to have specimens for hrHPV testing collected by a clinician rather than self-collected sampling. When directly comparing the two self-collection devices, most women preferred the self-collection Evalyn brush stored “dry” to the Viba brush stored in liquid media. Women strongly agreed that both self-collection brushes were comfortable during use, and the instructions easy to follow. For both self-collection approaches, most women somewhat agreed that they were concerned about performing self-collection properly.

Women in several studies from the US and Europe reported a preference for self-collection compared to clinician-collection for hrHPV specimen collection [4, 19, 20]. A meta-analysis including eight studies from Europe and two from North America also reported that, among women who did not routinely screen for cervical cancer, compliance was better when using self-collected genital specimens as initial screening as opposed to pap smears [4]. In contrast, one study from Cameroon reported that while women found self-collection more comfortable and less embarrassing, a greater proportion of women still preferred to have genital specimens collected by a clinician (62% vs. 29%, *p* < 0.001) [21]. In this African population, women reported greater concerns about the reliability of results from self-collected specimens as compared to clinician-collected specimens (59% vs. 1%). Our results suggest that Kenyan women may have similar concerns about the reliability of self-collected specimens. When asked, “Were you concerned about using the cytobrushes properly?” most of the women’s response was “agreed somewhat” (Table 4). This is in contrast to their responses on questions pertaining to the specific characteristics of the brushes, which were consistently very favorable. Most women, for example, felt that the self-collection brushes were comfortable to use, and instructions for self-collection easy to follow.

While the overall rating for both cytobrushes was positive, the Evalyn cytobrush was generally more preferred. Importantly, the majority of women in this study who reported greater preference for the Evalyn brush also found the cytobrush more comfortable to insert, and reported greater fears of hurting themselves with the Viba cytobrush. These two may be explained by the difference in physical characteristics of the Evalyn cytobrush. The cytobrush has a plunger, which allowed the women to pull down the bristles into the transparent casing at the point of insertion. The Evalyn brush also has wings which marks the furthest point the cytobrush can be inserted into the vagina. It is possible that these two characteristics may contribute to the greater comfort and less fears of hurting themselves when using the Evalyn brush. Additionally, women did not have to detach the tip of the Evalyn brush and insert into the liquid transport media and merely replaced the plastic cap after self-collection as the cytobrush was stored and transported dry. The finding that women reported a preference for one cytobrush over the other for self-collection is important for the potential scale-up of self-collection programs in this region. However, it is important to note that it is unknown whether women’s preference for the Evalyn brush would have a meaningful impact on self-collection program coverage if only an alternative like the Viba brush were offered.

It is interesting to note that while findings from our study and the Cameroonian study [21] agree, the study populations are distinctly different. Our study was conducted in female sexual workers at a notably high risk of infection with HIV and STIs, who regularly attended clinic and had frequent pelvic examinations. In contrast, the study from Cameroon was conducted in general population with limited exposure to pelvic examinations. The parallel findings from these two distinct populations of African women may point towards a general preference for clinician-collected genital specimens among women from this region, although more research is warranted to confirm these findings.

Our study has several strengths. Few other studies in sub-Saharan Africa have evaluated women’s preference between different methods of genital specimen-collection for hrHPV testing. East Africa contributes a significant burden of invasive cervical cancer globally, and reports one of the lowest proportion of reproductive-aged women having been screened for cervical cancer [22]. In addition, our study was conducted in a cohort of women at a particularly high risk for hrHPV and high-grade cervical lesions. Interventions aimed at improving hrHPV testing in this population are, therefore, likely to be of great benefit in terms of reducing preventable cervical cancer and associated mortality.

One important limitation of our study was that self-collection of genital specimens was conducted in a clinic setting. Women had to present to a health facility to conduct the self-test. Part of the challenge of cervical cancer screening in East Africa is poor access to health facilities. It is plausible that women would appreciate self-collection more if they were offered the additional convenience of self-collection at home or in another non-clinical setting. Another limitation was that this study was conducted in a group of women who were part of an ongoing cohort study in which pelvic examinations with collection of genital specimens for STI screening is routine. It is possible that the women in this population have grown more comfortable with clinical examinations compared to women in the general population.

## Conclusion

In conclusion, our findings suggest that high-risk women in sub-Saharan Africa may prefer clinician-collection, rather than self-collection, for hrHPV testing. Areas with inadequate healthcare infrastructure, especially those with few trained clinicians to conduct pelvic examinations, would benefit greatly from scale-up of self-collection for cervical cancer screening. In this regard, education about the validity, safety and ease of self-collected specimens for hrHPV detection will be important to enhance uptake of self-collection of genital specimens for cervical cancer screening.

## Abbreviations

CI: Confidence interval
CIN: Cervical intraepithelial neoplasia
HIV: Human immunodeficiency virus
HrHPV: High-risk human papillomavirus
Pap: Papanicolau smear
STI: Sexually transmitted infections
