# Correction to: Preference of specimen collection methods for human papillomavirus detection for cervical cancer screening: a cross-sectional study of high-risk women in Mombasa, Kenya

**DOI:** 10.1186/s12978-019-0812-8

**Published:** 2019-10-30

**Authors:** Griffins O. Manguro, Linnet N. Masese, Kishor Mandaliya, Susan M. Graham, R. Scott McClelland, Jennifer S. Smith, Vernon Mochache

**Affiliations:** 1 Kenyatta National Hospital, P.O. Box 91109, Mombasa, 80103 Kenya; 2 Department of Epidemiology, University of Washington, P.O Box 357236, Seattle, WA 98195 USA; 3 Pathcare Laboratories, Mombasa, Kenya; 4 Departments of Epidemiology, Global Health, and Medicine, University of Washington, P.O Box 359909, Seattle, WA USA; 5 Institute of Tropical and Infectious Diseases, University of Nairobi, Nairobi, Kenya; 6 University of North Carolina at Chapel Hill, Chapel Hill, NC USA; 7 Lineberger Cancer Center, Chapel Hill, NC USA; 8 Partnership for Advanced Clinical Education (PACE), Kamilisha, Kenya; 9 Center for International Health, Education, and Biosecurity (CIHEB), Institute of Human Virology, University of Maryland School of Medicine, KREP Centre, 6th Floor, Wood Avenue, Kilimani, Nairobi, P.O. Box 495, Nairobi, 00606 Kenya

**Correction to: Reprod Health**

**http://dx.doi.org/10.1186/s12978-018-0651-z**

Following publication of the original article [1], we have been notified that another author should be added to the team of authors. The Name and affiliation details are below.

Vernon Mochache; email: VMochache@mgic.umaryland.edu;

Program Director, Partnership for Advanced Clinical Education (PACE) Kamilisha - Kenya.

Center for International Health, Education, and Biosecurity (CIHEB).

Institute of Human Virology, University of Maryland School of Medicine, KREP Centre, 6th Floor, Wood Avenue, Kilimani, Nairobi, P.O. Box 495–00606, Nairobi, Kenya.
